# Contribution of the CYP51A Y119F Mutation to Azole Resistance in *Aspergillus flavus*

**DOI:** 10.3390/jof11110798

**Published:** 2025-11-10

**Authors:** Yabin Zhou, Yue Wang, Alexey A. Grum-Grzhimaylo, Martin Meijer, Bart Kraak, Zhengwen Li, Jos Houbraken

**Affiliations:** 1 Westerdijk Fungal Biodiversity Institute, Uppsalalaan 8, 3584 CT Utrecht, The Netherlands; chouyabin@163.com (Y.Z.); a.grum@wi.knaw.nl (A.A.G.-G.); m.meijer@wi.knaw.nl (M.M.); b.kraak@wi.knaw.nl (B.K.); 2 Cell Biology-Inspired Tissue Engineering, Institute for Technology-Inspired Regenerative Medicine, Maastricht University, Universiteitssingel 40, 6200 MD Maastricht, The Netherlands; yue.wang@maastrichtuniversity.nl; 3 School of Pharmacy, Chengdu University, Chengluo Avenue 2025, Chengdu 610106, China; lizhengwen@cdu.edu.cn

**Keywords:** *Aspergillus flavus*, azole resistance, CYP51A, site-directed mutagenesis, protein structure

## Abstract

*Aspergillus flavus* is both an agricultural and clinical pathogen, notable for its ability to contaminate crops with aflatoxins and cause invasive aspergillosis. The increasing emergence of azole resistance in *A. flavus* poses a serious challenge to food safety and human health. Although mutations in ergosterol biosynthesis genes have been reported in resistant isolates, their functional contributions remain largely unvalidated. In this study, we investigated the role of the CYP51A Y119F mutation in azole resistance. Site-directed mutants were generated using PCR-based gene editing, and their susceptibility to antifungal agents was assessed through Clinical and Laboratory Standards Institute broth microdilution and agar diffusion assays. The Y119F mutation reduced susceptibility specifically to voriconazole and isavuconazole, while susceptibility to itraconazole and posaconazole remained unchanged. To explore the structural basis of this phenotype, molecular dynamics simulations were performed. The mutant protein exhibited greater fluctuations and reduced conformational stability compared to the wild-type enzyme. Tunnel analysis further indicated that the Y119F substitution caused narrowing and shortening of the main access tunnels to the heme-binding pocket, likely impairing azole access and binding. The combined biochemical and structural analyses suggest that Y119F represents a primary resistance-conferring mutation that modifies the structural dynamics of CYP51A.

## 1. Introduction

*Aspergillus flavus* is a widely distributed fungal species with significant implications for both agriculture and human health. It is an opportunistic pathogen capable of infecting oilseed crops such as peanuts, corn, and cottonseed, as well as immunocompromised individuals. One of its most concerning attributes is its ability to produce toxic metabolites, including aflatoxins, a class of mycotoxins known for their severe health and economic consequences. Among these, aflatoxin B1 is the most potent, primarily targeting the liver and contributing to chronic diseases such as hepatocellular carcinoma [1]. Acute exposure to high concentrations of aflatoxins can lead to aflatoxicosis, a potentially fatal condition characterized by severe liver damage, with some cases resulting in death [2]. Aflatoxin contamination of crops poses a major economic threat, as agricultural commodities exceeding regulatory limits are often destroyed or substantially devalued. In the United States alone, economic losses due to aflatoxin-contaminated corn have been estimated to range from $52.1 million to $1.68 billion annually, with even greater losses anticipated as climate change creates conditions that favor aflatoxin production [3]. The situation is particularly dire in developing countries, where regulatory enforcement is often inadequate due to limited resources and infrastructure. As a result, approximately 4.5 billion people worldwide are at risk of chronic aflatoxin exposure, leading to increased rates of liver disease and other long-term health complications [4].

Beyond its impact on agriculture, *A. flavus* is also a clinically significant pathogen. Invasive aspergillosis (IA), a life-threatening fungal infection primarily affecting immunocompromised individuals, has been increasing in incidence worldwide. Each year, IA affects over 2.1 million people, with an exceptionally high mortality rate of 85.2% [5]. While *Aspergillus fumigatus* remains the leading cause of IA, *A. flavus* is the second most commonly isolated species, responsible for approximately 15–20% of infections [6]. Notably, in arid and high-temperature regions such as the Middle East, Africa, and Southeast Asia, *A. flavus* often surpasses *A. fumigatus* as the predominant pathogenic species, likely due to its thermotolerance and drought tolerance [7]. IA caused by *A. flavus* is associated with severe clinical outcomes, contributing to an estimated 300,000 deaths globally each year.

Azoles, a widely used class of antifungal drugs, are increasingly facing reduced efficacy due to the emergence of resistance in various fungal pathogens [8]. Studies have shown that resistance to voriconazole leads to a 21% decrease in survival rates for patients with IA, underscoring the critical impact of resistance on patient outcomes [9]. Azole resistance in *Aspergillus* species is a growing concern. For instance, in the Netherlands, the frequency of azole-resistant *A. fumigatus* increased from 7.6% in 2013 to 14.7% in 2018 [10]. Similarly, a ten-year study in Denmark reported a rise in azole resistance among patients with cystic fibrosis, from 4.5% in 2007–2009 to 10.5% in 2018 [11]. While the mechanisms of azole resistance in *A. fumigatus* have been extensively studied [12], research on azole resistance in *A. flavus* remains limited. *A. flavus* is abundant, and azole fungicides are commonly used in agriculture to protect crops from fungal contamination, creating scope for resistance selection. Azole-resistant *A. flavus* strains have been isolated from maize fields treated with azole fungicides [13]. A study in Vietnam found that over 85% of *A. flavus* isolates from environmental sources were resistant to at least one medical azole [6].

The *cyp51* genes encode key enzymes involved in ergosterol biosynthesis and are the primary targets of azole antifungal drugs. Different *Aspergillus* species harbor varying numbers of *cyp51* paralogs in their genomes. *Aspergillus fumigatus*, *Aspergillus terreus*, and *Aspergillus niger* each possess two paralogs (*cyp51A* and *cyp51B*), whereas *A. flavus* contains three (*cyp51A*, *cyp51B*, and *cyp51C*) [14]. In *A. fumigatus*, the TR34/L98H and TR46/T298A/Y121F mutations in the *cyp51A* gene are the most common mutations associated with azole resistance. Several *cyp51A* mutations have also been identified in azole-resistant *A. flavus* strains, but their functional roles remain unvalidated [15,16,17,18]. In our previous research, we detected a CYP51A Y119F mutation in an azole-resistant *A. flavus* strain isolated from a patient [18]. In this study, we aim to validate the functional role of the CYP51A Y119F mutation in azole resistance in *A. flavus*.

## 2. Materials and Methods

### 2.1. Alignment of CYP51A in Different Fungi

Deduced proteins sequence of *A. flavus* E15 CYP51A (NCBI accession number UKG18706), *Aspergillus oryzae* DIA-ITDAM CYP51A (NCBI accession number WLV76458), *A. niger* ATCC 1015 CYP51A (NCBI accession number AEK81582), *A. terreus* IFO 6365 CYP51A (NCBI accession number GFF17748), *A. fumigatus* Af293 CYP51A (NCBI accession number EAL90099), *Penicillium digitatum* PdW03 CYP51A (NCBI accession number QQK40601), *Rhizopus arrhizus* ATCC 11886 CYP51A (NCBI accession number AWM98458), *Mucor circinelloides* 35-18HB14-257 CYP51A (NCBI accession number WAU48787), *Fusarium graminearum* 16D1 CYP51A (NCBI accession number AFN66169), *Fusarium cerealis* NRRL 13721 CYP51A (NCBI accession number AFN66168), *Fusarium austroamericanum* NRRL 28718 CYP51A (NCBI accession number AFN66166), *Fusarium vorosii* 67C1 CYP51A (NCBI accession number AFN66167), *Neurospora crassa* OR74A CYP51A (NCBI accession number EAA34813), *Candida albicans* SC5314 ERG11 (NCBI accession number AOW29509), *Nakaseomyces glabratus* (formerly *Candida glabrata*) ATCC 2001 ERG11 (NCBI accession number QHS65513), *Pichia kudriavzevii* (formerly *Candida krusei*) ATCC 6258 ERG11 (NCBI accession number AMR44147), *Candida parapsilosis* ATCC 22019 ERG11 (NCBI accession number ACT67904), *Candida tropicalis* ATCC 750 ERG11 (NCBI accession number AMR44151), *Saccharomyces cerevisiae* CBS 8340 ERG11 (NCBI accession number QHB08993), *Rhodotorula mucilaginosa* KR ERG11 (NCBI accession number KAG0657364), *Trichosporon asahii* OMU239 ERG11 (NCBI accession number ADN44281), *Cryptococcus neoformans* H0058-I-3095 ERG11 (NCBI accession number WIV42398), *Cryptococcus gattii* WM276 ERG11 (NCBI accession number AEQ63274), and *Cryptococcus deuterogattii* R265 ERG11 (NCBI accession number AEQ63272) were used for alignment by MEGA 11.

### 2.2. Construction of CYP51A Y119F Mutants

The CYP51A Y119F (Tyr119 to Phe) mutation was introduced using PCR-driven overlap extension. Two overlapping fragments of the *cyp51A* gene were amplified from *A. flavus* NRRL 3357-5 genomic DNA using primer pairs F1/R1 and F2/R2, with the mutation incorporated in primers R1 and F2. These fragments were fused to generate a 1.6 kb PCR product carrying the mutated *cyp51A* sequence. For the wild-type control, a cyp51A fragment was amplified using primer pair F1/R2 from *A. flavus* NRRL 3357-5 DNA. The *pyrG* selection marker was amplified from plasmid pFC330 [19] using primer pair F3/R3, while the 3′-flanking region of *cyp51A* was amplified using primer pair F4/R4 from *A. flavus* NRRL 3357-5 DNA. The final mutated cassette was assembled by amplifying the cyp51A mutant sequence, *pyrG*, and the 3′-flanking region using primer pair F5/R5 (Figure S1). A wild-type cassette, lacking mutations in *cyp51A*, was similarly constructed using the wild-type *cyp51A* sequence.

The preparation and transformation of *A. flavus* protoplasts were performed as previously described [20]. *A. flavus* NRRL 3357-5 (*pyrG* mutant of NRRL 3357) was used as recipient strain. Each transformation used 1 μg of either the mutated or wild-type cassette. Transformants were purified by two rounds of single-colony streaking on Czapek’s agar (CZA) plates. Genomic DNA from putative mutants was extracted and used as a PCR template. Correct mutants were verified by amplifying the *cyp51A* gene with primer pair F6/R6 (Figure S2), followed by Sanger sequencing (Figure S3). The mutants with CYP51A Y119F mutation and wild type CYP51A were named as P51A^Y119F^ and P51A^WT^, respectively. All the primers used in this study were listed in Table S1.

### 2.3. Antifungal Susceptibility Testing

The Clinical and Laboratory Standards Institute (CLSI) M38-A3 broth microdilution reference method was used to evaluate susceptibility to itraconazole, voriconazole, posaconazole, isavuconazole, caspofungin, micafungin, anidulafungin, and amphotericin B [21]. Antifungal drugs were purchased from Merck company and obtained as powders. The drugs were tested in the following final concentration ranges: 0.03–16 mg/L for itraconazole, voriconazole, posaconazole, isavuconazole and amphotericin B; and 0.008–4 mg/L for caspofungin, micafungin, and anidulafungin. Minimum inhibitory concentrations (MICs) or minimum effective concentrations (MECs) were determined after incubation at 35 °C for 48 h. MIC endpoints were defined as the lowest drug concentration that completely inhibited growth, while MEC endpoints were determined as the lowest drug concentration that resulted in small, rounded, compact hyphal forms compared to the filamentous hyphal growth observed in the control well. Azole susceptibility was further assessed using agar diffusion with paper disks, following CLSI M51-A guidelines [22]. All antifungal susceptibility tests were performed in triplicate on three separate days. *Aspergillus fumigatus* ATCC MYA-3626 and *C. parapsilosis* ATCC 22019 were included as quality control strains.

### 2.4. Morphological Analysis

Strains were inoculated on potato dextrose agar (PDA) using a 2 μL inoculum of 10^6^ spores/mL in triplicate. Plates were incubated for 4 days at 25 °C, 30 °C, 37 °C, and 40 °C. Zone diameters were measured using a ruler, and sporulation was assessed visually.

### 2.5. In Silico Protein Structure Analysis

The wild-type *A. flavus* CYP51A protein (WT) sequence and three-dimensional (3D) structure were obtained from the UniProt database [23]. The sequence of mutant CYP51A protein (MU) was generated in PyMOL 2.5 [24] by replacing the tyrosine (Y) at position 119 with phenylalanine (F). The initial three-dimensional (3D) structure was predicted using AlphaFold 3 [25], which provided a high-confidence model.

### 2.6. Energy Minimization and Molecular Dynamics Simulations

To refine the structures, both WT and MU proteins underwent energy minimization using GROMACS 2023.1 [26] with the AMBER99SB-ILDN force field [27]. The SPC/E water model was used for solutions, and counterions (Na^+^/Cl^−^) were added to neutralize the system [28]. Energy minimization was performed using the steepest descent algorithm until the maximum force was below 1000 kJ/mol/nm. Subsequently, molecular dynamics (MD) simulations were carried out for 500 ns at 300 K using the V-rescale thermostat [29]. Pressure coupling was maintained with the Parrinello–Rahman barostat [30]. Bond lengths were constrained using the LINCS algorithm [31], and long-range electrostatic interactions were treated with the Particle Mesh Ewald (PME) method [32].

The structural dynamics of both WT and MU proteins were evaluated using three key parameters with the built-in analysis tools of GROMACS [26]. Root Mean Square Deviation (RMSD) of the Cα atoms was calculated to assess the overall conformational stability of the proteins throughout the simulation trajectories. Root Mean Square Fluctuation (RMSF) was computed to examine residue-level flexibility, focusing on backbone atoms to identify regions with notable fluctuations. Hydrogen bond analysis was performed to quantify intra-protein hydrogen bonds over time, providing insights into the stability and integrity of the proteins’ secondary and tertiary structures [33].

### 2.7. Tunnels Analysis

Tunnel and channel detection from the protein surface to the active site (HEME group) was performed using CAVER 3.0 [34]. The iron atom (Fe) of the HEME group was defined as the starting point for tunnel calculations. Key parameters were set as follows: probe radius of 0.9 Å, shell radius of 3.0 Å, shell depth of 4.0 Å, and clustering threshold of 3.5 Å. CAVER identified potential access pathways connecting the protein surface to the buried HEME center. The resulting tunnels were evaluated based on their length, curvature, and throughput score.

### 2.8. Statistical Analysis

Data analysis and graph generation were performed using GraphPad Prism 8 software. Results are presented as the mean ± standard deviation (SD). Statistical significance was assessed using Student’s *t*-test for comparisons between two groups. A *p*-value of < 0.05 was considered statistically significant.

## 3. Results

### 3.1. CYP51A Y119 Is a Conserved Amino Acid in Fungi Except Mucorales

**Figure 1:**
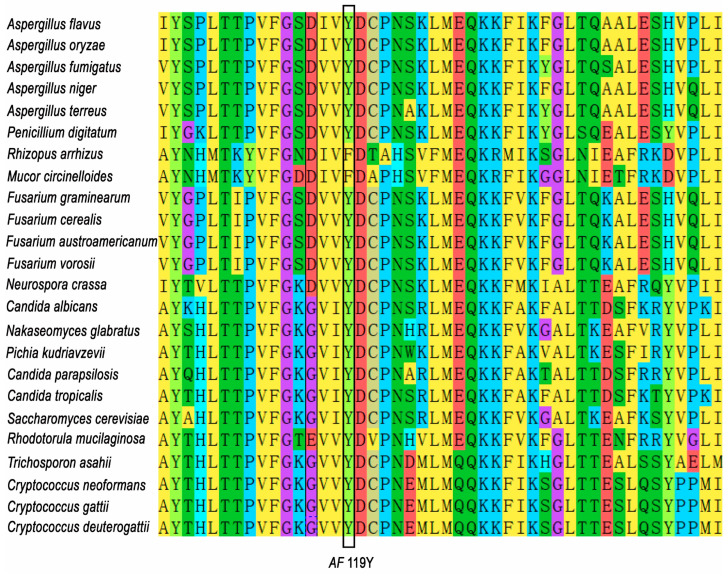
Alignment of position Y119F in *A. flavus* in 24 fungal species. In Y119, 22 out of 24 species also have tyrosine (Y) at that position. The other 2 Mucorales species have a phenylalanine (F) on this position.

Sequence alignments of CYP51A across diverse fungal species reveal that the tyrosine residue at position 119 (Y119) is highly conserved among most fungi (Figure 1). In filamentous fungi such as *A. flavus*, *A. fumigatus*, *A. niger*, *P. digitatum*, and *F. graminarum*, Y119 is consistently present. Similarly, yeasts including *C. albicans*, *S. cerevisiae*, and *Cry. neoformans* retain a conserved tyrosine residue at this position. This conservation suggests a critical structural or functional role for Y119 in the enzymatic activity of CYP51A. However, in members of the order Mucorales, such as *R. arrhizus* and *M. circinelloides*, Y119 is replaced by phenylalanine.

### 3.2. CYP51A Y119F Mutation Shows Decreased Susceptibility to Voriconazole and Isavuconazole but Maintained Susceptibility to Itraconazole and Posaconazole in A. flavus

**Figure 2:**
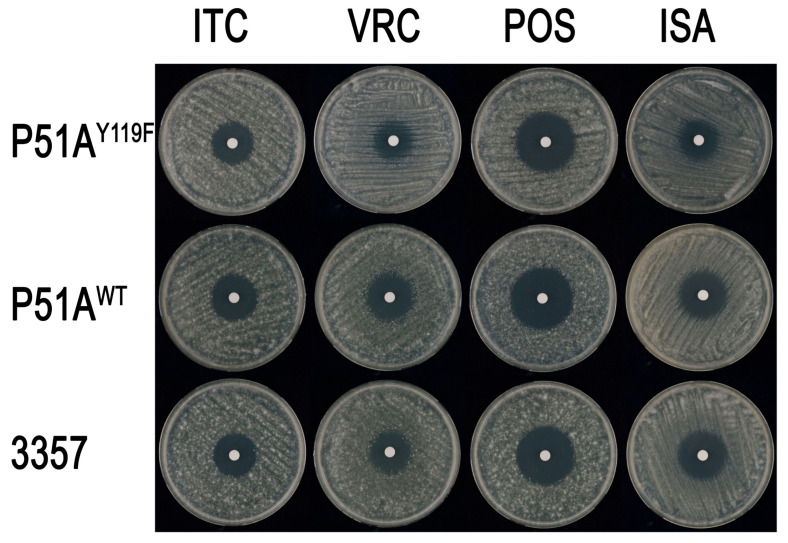
Antifungal susceptibility testing using the disk diffusion method for *A. flavus* P51A^Y119F^, P51A^WT^ and NRRL 3357.

Susceptibility testing revealed reduced susceptibility to voriconazole and isavuconazole (Table 1 and Figure 2) but not to itraconazole, posaconazole, caspofungin, micafungin, anidulafungin, or amphotericin B in CYP51A Y119F mutant. This phenotype was reproduced in the transformant P51A^Y119F^, compared to P51A^WT^ and NRRL 3357. Voriconazole susceptibility decreased by three dilution steps, whereas isavuconazole susceptibility decreased by only one. These results confirm that the CYP51A Y119F mutation in *A. flavus* reduces susceptibility to voriconazole and isavuconazole.

### 3.3. CYP51A Y119F Mutation Does Not Cause Fitness Cost in A. flavus

**Figure 3:**
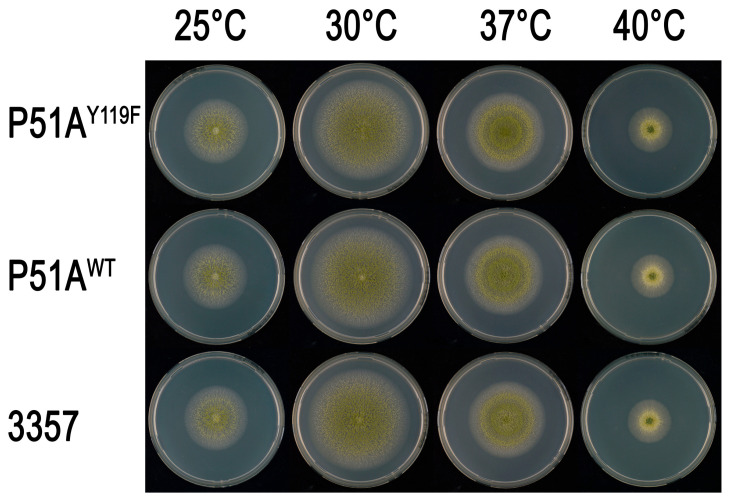
Colonies growth of *A. flavus* P51A^Y119F^, P51A^WT^ and NRRL 3357 on PDA for 4 days at 25 °C, 30 °C, 37 °C, and 40 °C.

**Figure 4:**
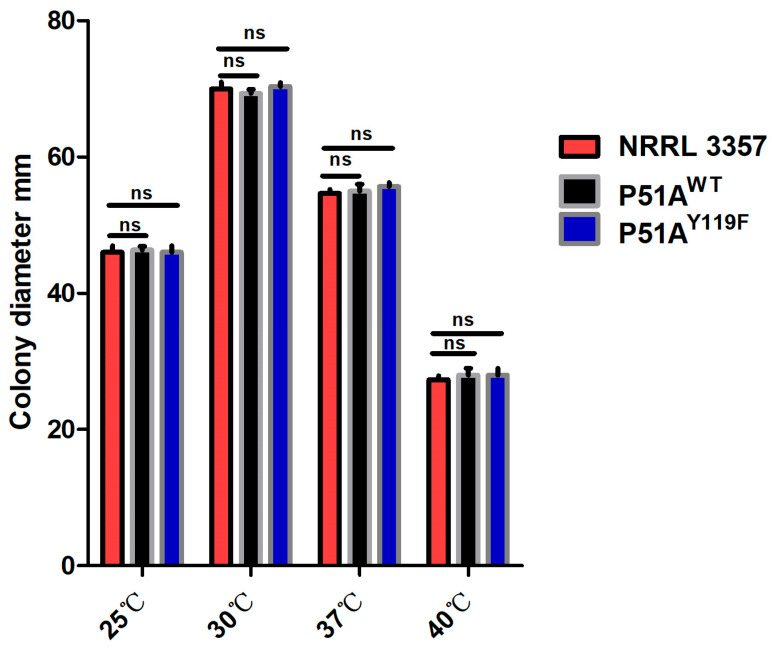
Growth measurements of of *A. flavus* P51A^Y119F^, P51A^WT^ and NRRL 3357 on PDA for 4 days at 25 °C, 30 °C, 37 °C, and 40 °C. ns, *p* > 0.05.

After 4d incubation on PDA, all strains (P51A^Y119F^, P51A^WT^ and NRRL 3357) had similar colony diameters at the same temperature (25 °C, 30 °C, 37 °C and 40 °C) (Figure 3 and Figure 4). Sporulation also appeared comparable among the strains, indicating that the CYP51A Y119F mutation does not impose a fitness cost in *A. flavus*.

### 3.4. Structural Stability Is Changed by CYP51A Y119F Mutation

**Figure 5:**
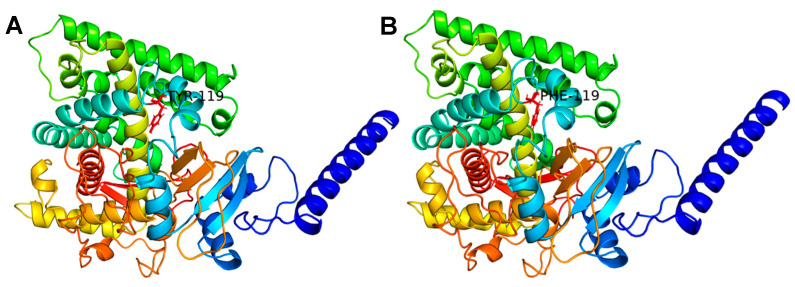
Optimized three-dimensional structures of wild-type (WT, (**A**)) and Y119F mutant (MU, (**B**)) CYP51A proteins after energy minimization. The initial structures were predicted using AlphaFold 3.0 and subsequently minimized with GROMACS. Final conformations were visualized using PyMOL.

To assess the impact of the mutation on the structural stability of CYP51A, both WT and MU proteins underwent energy minimization. The resulting final confirmations are shown in Figure 5. The potential energy of the WT system (−1.75821 × 10^6^ kJ/mol) was slightly lower than that of the MU system (−1.75809 × 10^6^ kJ/mol), indicating a marginally more stable configuration (Table 2). The small difference in potential energy (~120 kJ/mol) suggests that the mutation has a minimal effect on the overall conformational stability of the protein. However, the total energy exhibited a substantial shift—from a positive value in the WT to a negative value in the MU—with a difference of approximately 2.8 × 10^6^ kJ/mol. This notable change may reflect a redistribution of internal energy, possibly associated with altered binding free energy or the release of internal strain resulting from the mutation.

**Figure 6:**
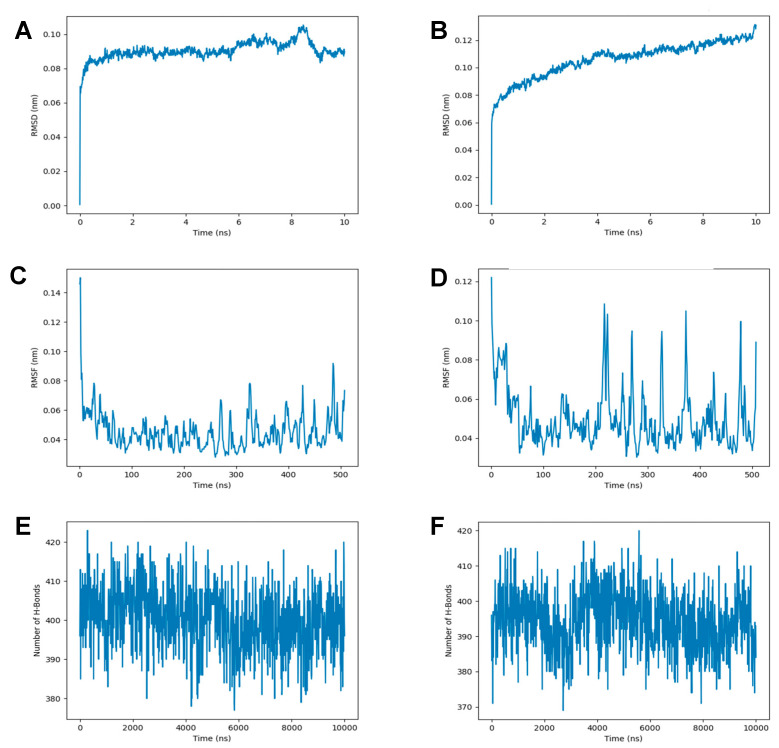
Dynamic behavior analysis of WT and MU CYP51A proteins over 500 ns MD simulations: (**A**,**B**) Root Mean Square Deviation (RMSD) of Cα atoms; (**C**,**D**) Root Mean Square Fluctuation (RMSF) per residue; (**E**,**F**) Number of intra-protein hydrogen bonds over time.

Following energy minimization, molecular dynamics simulations were conducted for 500 ns. RMSD analysis (Figure 6A,B) showed that the WT protein reached equilibrium within approximately 1 ns, maintaining a stable average RMSD of ~0.09 nm. In contrast, the MU protein displayed a gradual increase in RMSD, reaching ~0.12 nm by 10 ns and exhibiting higher fluctuations throughout the simulation, suggesting reduced overall conformational stability. RMSF profiles (Figure 6C,D) indicated that while both proteins showed generally low flexibility, specific regions—particularly around residue 119 and the heme-accessible channel—demonstrated increased fluctuations in the mutant. This implies that the mutation locally disrupts structural rigidity, potentially affecting substrate access or binding. Hydrogen bond analysis (Figure 6E,F) further supported these observations: the WT protein maintained approximately 400 intra-protein hydrogen bonds with minimal variation, whereas the MU protein exhibited a slightly lower average number of hydrogen bonds and greater fluctuation amplitude. These findings indicate weakened internal hydrogen bonding and compromised tertiary structure stability in the MU protein, which may contribute to altered function or reduced drug susceptibility.

### 3.5. Tunnel Geometry Is Changed by CYP51A Y119F Mutation

**Figure 7:**
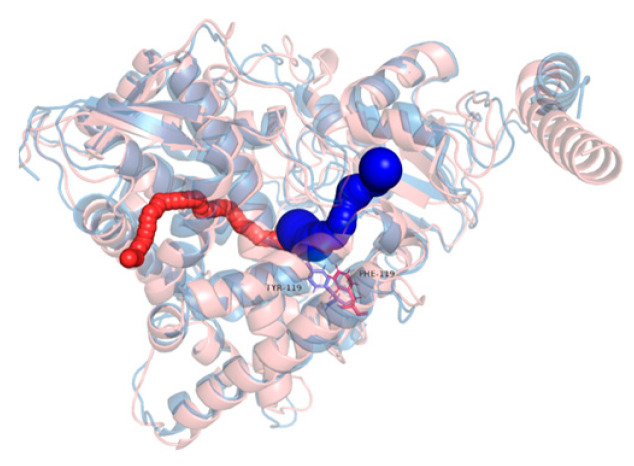
Visualization of the dominant substrate access tunnel in WT (Blue) and MU (red) CYP51A proteins, calculated by CAVER 3.0.

To assess whether the Y119F mutation affects drug access pathways, tunnel analysis was conducted on representative structures of both the WT and MU proteins using CAVER 3.0. The primary tunnels connecting the protein surface to the heme group were evaluated based on bottleneck radius, tunnel length, curvature, and throughput score (Table 3; Figure 7).

In the WT protein, the dominant tunnel exhibited a larger bottleneck radius (1.72 Å), greater length (23.31 Å), and a higher throughput score (0.6162), suggesting a more favorable geometry for substrate access. In contrast, the corresponding tunnel in the mutant displayed a narrower bottleneck (1.34 Å), shorter length (18.61 Å), and involved more bottleneck residues, potentially indicating a more obstructed or dynamically unstable path.

These results indicate that the Y119F mutation induces subtle structural rearrangements that alter the geometry of access tunnels to the active site, which may in turn affect drug binding affinity or catalytic efficiency.

## 4. Discussion

Our study demonstrates that the CYP51A Y119F mutation in *A. flavus* reduces susceptibility to voriconazole and isavuconazole while maintaining susceptibility to itraconazole and posaconazole. This finding contributes to the growing understanding of azole resistance mechanisms in *A. flavus*, which remain underexplored compared to *A. fumigatus*. Given the clinical and agricultural importance of *A. flavus*, our findings highlight the need for increased surveillance and molecular characterization of azole-resistant isolates.

Sequence analysis revealed that Y119 is highly conserved across diverse fungal species, suggesting an important role in CYP51A enzymatic function. Notably, in Mucorales species, phenylalanine (F) replaces tyrosine (Y) at this position, a mutation potentially linked to intrinsic voriconazole resistance in this fungal group [35]. However, its impact in *A. flavus* was previously unknown. Our study is the first to provide experimental evidence linking Y119F to azole resistance in this species. Previous research has shown that specific mutations in CYP51 proteins across different fungal species can alter azole binding affinity, often leading to antifungal resistance. Well-documented examples in *A. fumigatus* CYP51A include G54A, G54W, P216L, M220V/K/T, and G448S, all of which confer azole resistance [36]. The strong conservation of Y119 suggests its importance in maintaining CYP51A structure and function, which may explain why its mutation affects azole susceptibility. Homologous Y119F mutations in other fungal species have demonstrated similar effects on azole resistance. For instance, the Y121F mutation in *A. fumigatus* is directly associated with increased voriconazole MICs [37]. The Y132F mutation has been identified in azole-resistant *C. albicans* and is also present in resistant isolates of *Candida auris* and *C. parapsilosis* [38,39,40]. Similarly, the Y145F mutation in *Cry. neoformans* and the Y136F mutation in *Histoplasma capsulatum* are linked to reduced voriconazole susceptibility [41,42]. Additionally, the Y137F mutation in *Mycosphaerella graminicola* and the Y136F mutation in *Uncinula necator* confer resistance to triadimenol, a triazole structurally more similar to voriconazole than to the long-tailed triazoles itraconazole and posaconazole [43,44].

Antifungal susceptibility testing confirmed that the CYP51A Y119F mutation reduces susceptibility to voriconazole and isavuconazole but does not affect susceptibility to itraconazole or posaconazole. These findings align with previous studies in *A. fumigatus*, where isolates harboring the sole Y121F mutation exhibit reduced susceptibility to voriconazole but retain susceptibility to itraconazole and posaconazole [45]. The differential resistance patterns suggest that voriconazole and isavuconazole interact with a CYP51A region directly influenced by the Y119F mutation, whereas itraconazole and posaconazole may engage in additional stabilizing interactions that mitigate the impact of this mutation. Structurally, this phenomenon likely reflects differences in azole side-chain architecture. Itraconazole and posaconazole are long-tailed azoles, capable of forming extended hydrophobic contacts within the CYP51 substrate access channel. Voriconazole, by contrast, is a short-tailed azole, and isavuconazole has a medium-length tail [46]. The Y119F mutation therefore appears to primarily compromise the binding of short-tailed azoles such as voriconazole, moderately affect mid-length-tailed agents like isavuconazole, and exert minimal impact on long-tailed azoles including itraconazole and posaconazole.

Clinically, this has important implications, as strains carrying Y119F may remain susceptible to itraconazole or posaconazole even if resistant to voriconazole and isavuconazole. Notably, while the Y119F mutation significantly reduces voriconazole susceptibility, its effect on isavuconazole is less pronounced, decreasing susceptibility by only one dilution.

Structural and dynamic analyses indicate that the CYP51A Y119F mutation induces both structural destabilization and functional impairment. While the potential energy difference between wild-type (WT) and mutant (MU) proteins was minimal, MD simulations revealed higher RMSD, localized RMSF increases near residue 119 and the heme-access channel, and reduced, more variable intramolecular hydrogen bonding in MU. These perturbations suggest loss of local rigidity and compromised active-site architecture.

Tunnel analysis further demonstrated a narrower, shorter, and more obstructed access pathway in MU, consistent with reduced channel stability and substrate accessibility. Collectively, these findings support a mechanism in which Y119F confers resistance primarily through destabilizing dynamic regions critical for substrate entry, rather than altering intrinsic binding affinity. Experimental structural determination will be essential to confirm these conformational rearrangements.

The emergence of azole-resistant *A. flavus* poses a dual threat to both human health and agricultural biosecurity. Clinically, *A. flavus* is the second most common cause of invasive aspergillosis, and azole resistance has been associated with increased mortality rates [14]. Given that azole antifungals remain the frontline therapy for aspergillosis, the emergence of resistant isolates could compromise treatment outcomes. Our results suggest that patients infected with *A. flavus* harboring the CYP51A Y119F mutation may experience reduced efficacy of voriconazole and isavuconazole, potentially necessitating alternative therapeutic strategies such as lipid formulations of amphotericin B or combination antifungal therapy.

From an agricultural perspective, *A. flavus* is a major pathogen responsible for aflatoxin contamination in crops such as maize, peanuts, and cottonseed. Azole fungicides are widely used in agriculture to control fungal contamination; however, prolonged exposure to these compounds may drive the selection of resistant *A. flavus* strains. Previous studies have identified azole-resistant *A. flavus* isolates from agricultural environments [6], raising concerns about cross-resistance between environmental and clinical settings. The widespread use of azoles in both medicine and agriculture underlines the need for integrated resistance management strategies to mitigate the spread of resistant *A. flavus* strains.

One key finding of our study is that the CYP51A Y119F mutation does not impose a fitness cost on *A. flavus*. Strains carrying this mutation exhibited comparable colony growth and sporulation to wild-type strains under various temperature conditions. This is concerning from a public health perspective, as resistance mutations that do not impair fungal fitness are more likely to persist and disseminate in both clinical and environmental settings. In contrast, some azole-resistant *A. fumigatus* strains exhibit a fitness cost, which may restrict their prevalence in certain environments [47]. The absence of a fitness cost in Y119F *A. flavus* suggests that this mutation could become widespread if selection pressures favor its persistence, necessitating continuous monitoring of azole-resistant *A. flavus* isolates in clinical and agricultural settings.

## 5. Conclusions

Our study provides the functional validation of the CYP51A Y119F mutation in *A. flavus*, demonstrating its role in reduced susceptibility to voriconazole and isavuconazole. Notably, this is the first confirmed *cyp51A* gene mutation associated with azole resistance in *A. flavus*. These findings enhance our understanding of azole resistance mechanisms in *A. flavus* and highlight the need for increased surveillance of resistant strains. As azole resistance in *A. flavus* continues to emerge, collaboration among medical mycologists, agricultural scientists, and policymakers will be crucial in developing strategies to curb the spread of resistant strains while preserving the efficacy of azole antifungals.

## Figures and Tables

**Table 1:** Antifungal susceptibilities of strains in this study.

Strains	MIC/MEC (mg/L)								Disk Diameter (mm)			
	ITC	VRC	POS	ISA	AMB	CAS	MCF	AND	ITC	VRC	POS	ISA
P51A^Y119F^	1	4	0.125	1	1	0.06	≤0.008	≤0.008	26	19	37	27
P51A^WT^	1	0.5	0.125	0.5	1	0.06	≤0.008	≤0.008	26	25	37	31
NRRL 3357	1	0.5	0.125	0.5	1	0.06	≤0.008	≤0.008	26	25	37	31

ITC: Itraconazole, VRC: Voriconazole, POS: Posaconazole, ISA: Isavuconazole, AMB: Amphotericin B, CAS: Caspofungin, MCF: Micafungin, AND: Anidulafungin.

**Table 2:** Comparison of energy parameters between wild-type and mutant proteins based on molecular dynamics simulations.

Type	Energy Type	Energy (kJ/mol)	Error Est.	RMSD	Total Drift
WT	Potential Energy	−1,758,210	630	2972.99	−2699.73
	Total Energy	1,413,930	850	3978.78	−3519.45
MU	Potential Energy	−1,758,090	170	1640.31	−683.29
	Total Energy	−1,414,170	220	2107.31	−825.91

**Table 3:** Geometric parameters of the dominant access tunnel to the heme active site in WT and MU CYP51A proteins, calculated using CAVER 3.0. Parameters include bottleneck radius, tunnel length, curvature, key bottleneck residues, and throughput score.

Protein	Bottleneck Radius (Å)	Length (Å)	Curvature	Bottleneck Residues	Throughput Score
WT	1.7235	23.31	1.3108	105, 358, 487, 488	0.6162
MU	1.3369	18.61	1.1221	153, 156, 157, 179, 183, 460, 464	0.5787

## Data Availability

The original contributions presented in this study are included in the article. Further inquiries can be directed to the corresponding author.

## Supplementary Materials

The following supporting information can be downloaded at: https://www.mdpi.com/article/10.3390/jof11110798/s1, Figure S1: Strategy to construct CYP51A Y119F mutant; Figure S2: PCR verification of transformants; Figure S3: Sanger sequencing of transformants; Table S1: Primers used in this study.

## Abbreviations

The following abbreviations are used in this manuscript:

AMB	Amphotericin B
AND	Anidulafungin
CAS	Caspofungin
CLSI	Clinical and Laboratory Standards Institute
CZA	Czapek’s agar
IA	Invasive aspergillosis
ISA	Isavuconazole
ITC	Itraconazole
MCF	Micafungin
MD	Molecular dynamics
MEC	Minimum inhibitory concentration
MIC	Minimum effective concentration
MU	Mutant
PDA	Potato dextrose agar
PME	Particle Mesh Ewald
POS	Posaconazole
RMSD	Root Mean Square Deviation
RMSF	Root Mean Square Fluctuation
VRC	Voriconazole
WT	Wild type
