# Supplementary material for: Contribution of the CYP51A Y119F Mutation to Azole Resistance in Aspergillus flavus
Source: J Fungi (Basel). 2025 Nov 10;11(11):798. doi: 10.3390/jof11110798 (PMC12653524; doi:10.3390/jof11110798)
Supplement: Supplementary file 1 [file jof-11-00798-s001.zip › jof-3935966-supplementary.pdf]

SUPPLEMENTARY MATERIALS

TABLE S1 Primers used in this study

Name	Sequence (5'–3')
F1	TCCTTCACTCTCGTCAGCGCCTACGCGGCT
R1	*CTTTGAATTTGGACAATCAAACACAATGTCGGAGCCAAAG*
F2	*CTTTGGCTCCGACATTGTGTTTGATTGTCCAAATTCAAAG*
R2	*GTACACGAGGAATTACACTT*TCATGACTTTTCTGGGAAGC
F3	*GCTTCCCAGAAAAGTCATGA*AAGTGTAATTCCTCGTGTAC
R3	*AGGAAGGCTTTAATTAGGGC*GCGGAAAACATGGCATTACA
F4	*TGTAATGCCATGTTTTCCGC*GCCCTAATTAAAGCCTTCCT
R4	TGTCGCTTGCGTTGGTCGTTCAATCTCATT
F5	CCTAAATCTGTTGCGCCAGC
R5	CTGACCCAACGGTCCGTAAA
F6	TTGCATATCCGGGTGGTGTC
R6	CGGCGCTAACTATGGTTGACT

The italic parts in the primers were the complementary tails to the adaptor primer and the underlined base in the primers was mutation site

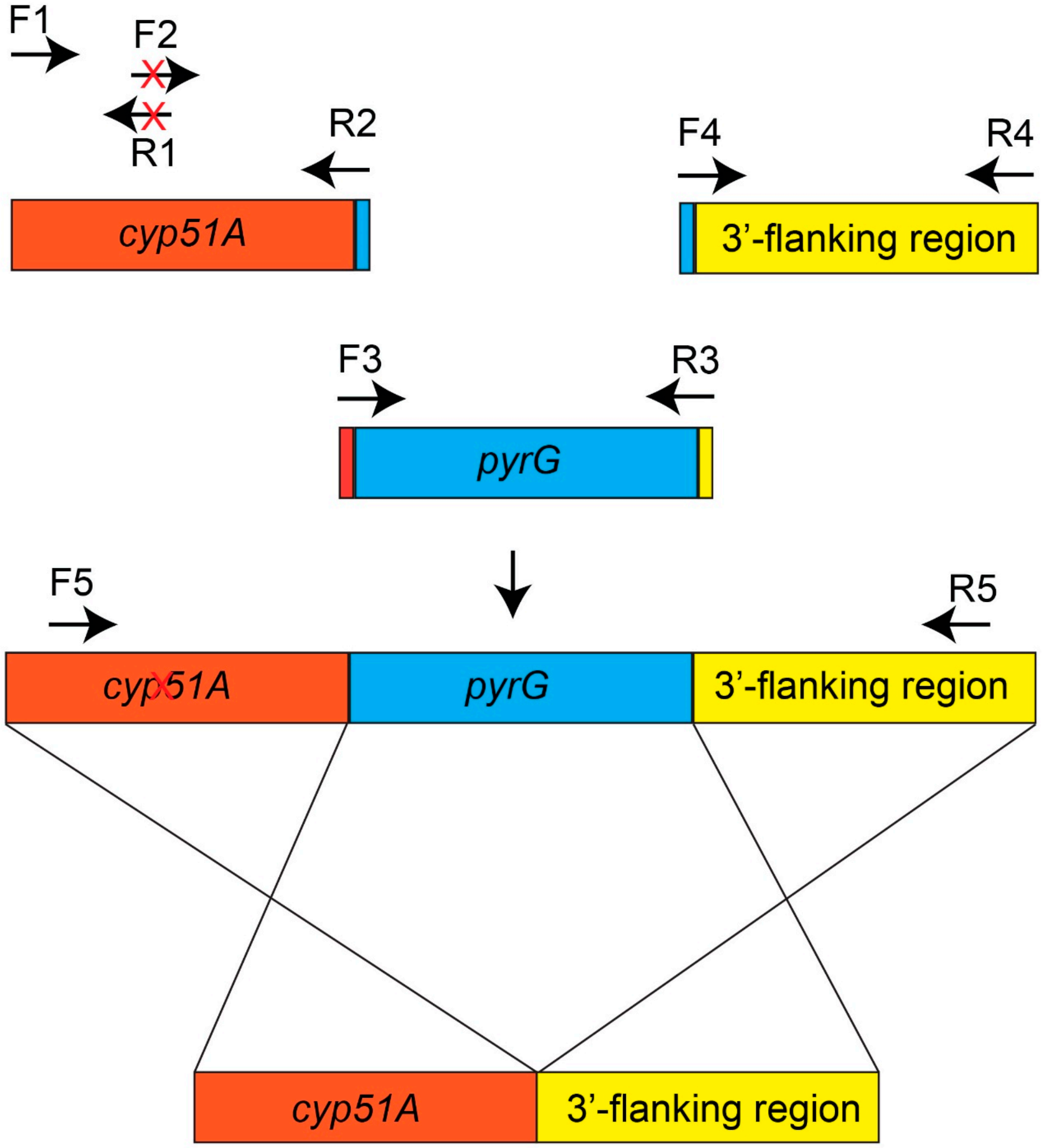

Figure S1 Strategy to construct CYP51A Y119F mutant

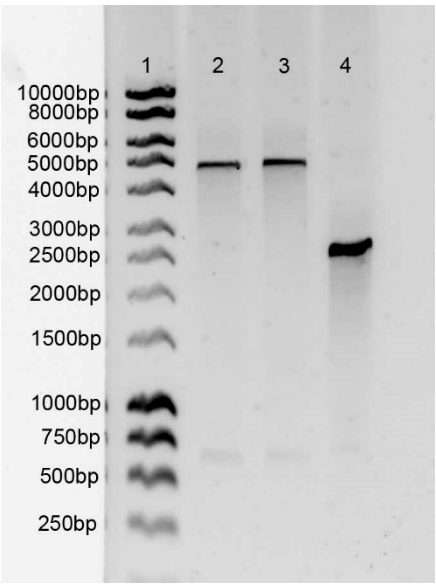

Figure S2 PCR verification of transformants. Lane 1, DNA marker. Lane 2, DNA amplified from P51A^{Y119F} by primers F6 and R6. Lane 3, DNA amplified from P51A^{WT} by primers F6 and R6. Lane4, DNA amplified from NRRL 3357 by primers F6 and R6. The PCR amplicon was approximately 2.8 kb in NRRL 3357 and about 4.7 kb in both P51A^{Y119F} and P51A^{WT} strains.

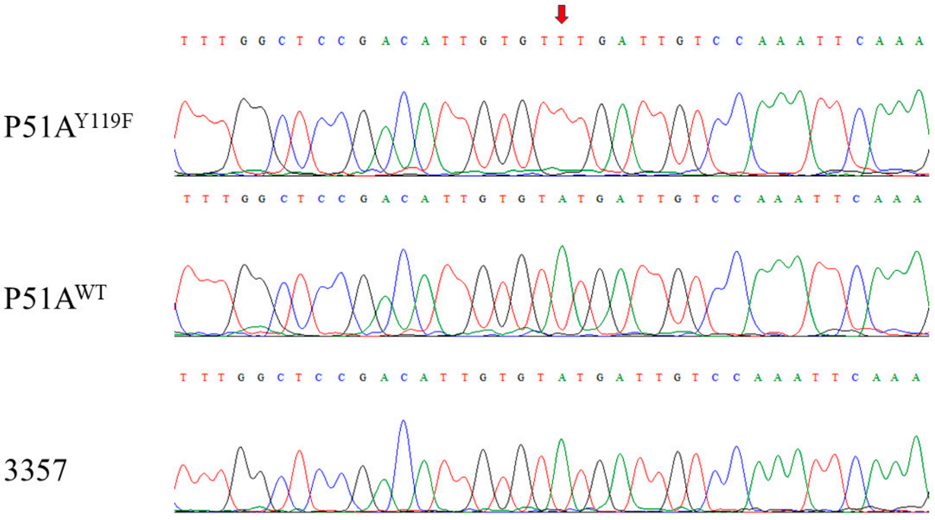

Figure S3 Sanger sequencing of transformants (arrowhead mutation site). Transformant P51A^{Y119F} was successfully incorporated the A423T nucleotide

substitution in the *cyp51A* gene, resulting in the Y119F amino acid change in the CYP51A protein.
